# Physico-Chemical, Textural and Sensory Evaluation of Emulsion Gel Formulated with By-Products from the Vegetable Oil Industry

**DOI:** 10.3390/gels9120964

**Published:** 2023-12-08

**Authors:** Ana Leahu, Sorina Ropciuc, Cristina Ghinea, Cristina Damian

**Affiliations:** Faculty of Food Engineering, Stefan cel Mare University of Suceava, 720229 Suceava, Romania; sorina.ropciuc@fia.usv.ro (S.R.); cristina.ghinea@fia.usv.ro (C.G.); cristinadamian@fia.usv.ro (C.D.)

**Keywords:** emulsion gel, vegetable oil by-products, sesame seeds cake, walnuts cake, sensory analysis, food applications

## Abstract

The aim of this study was to obtain low fat mayonnaise-like emulsion gels using sesame cake and walnut cake by-products resulting from vegetable oil extraction. The ingredients used to formulate the mayonnaise like emulsion gel samples were corn starch, sesame seed cake (SSC), walnuts seed cake (WSC), lemon juice, sunflower oil, mustard, sugar, salt, gelatin and water. Five different samples were prepared: one control lab sample (M) containing only corn starch and the other ingredients (without SSC and WSC), two samples (SO1 and SO2) with 2 and 4% of SSC (without corn starch and WSC) and two samples (WO1 and WO2) with 2 and 4% of WSC (without corn starch and SSC). Also, an egg-free commercial mayonnaise (CM) was purchased and used for comparison. Physicochemical (fat, protein, moisture, ash, carbohydrate, water activity, emulsion stability, viscosity, density and color), textural (hardness, adhesiveness, springiness, cohesiveness, gumminess and chewiness), and sensory (aspect, color, texture/firmness, flavor, taste and acceptability) attributes of all samples were investigated. The results showed that carbohydrate content decreased in all four seed cakes samples compared to the control sample, while protein and fat content increased in all seed cakes samples, with the largest increases observed in the sesame seed cake samples. It was observed that the CM sample has a carbohydrate content value close to that obtained for the M sample, while the protein content has the lowest value for the CM sample compared to all samples analyzed. The stability of the emulsion gels increased from 70.73% (control sample) to 83.64% for the sample with 2% addition sesame seed cake and to 84.09% for the 2% walnut cake added, due to the coagulation capacity of the added cakes. The type and concentration of oil seeds cake added in emulsion gels affected their textural properties such as hardness, adhesiveness, gumminess, and chewiness. The hardness and adhesiveness of low-fat mayonnaise-like emulsion gels samples decreased with the addition of oil seeds cake. However, the addition of by-products improved the sensory properties of emulsion gels. This study provided a theoretical basis for the food industry’s application of oilseed cakes, especially for the development of low-fat mayonnaise.

## 1. Introduction

Mayonnaise, a semisolid oil-in-water (O/W) emulsion [1], is a condiment consumed all over the world, used in various cold vegetable salads, or with fish and seafood, hamburgers, hotdogs, or used to garnish many other types of appetizers. It is also used as an ingredient in barbecue sauce and marinades, or to make dressings. Mayonnaise, one of the oldest and most used sauces in the world, is traditionally prepared with sunflower, corn, soybean and rapeseed oil mixed with egg yolks [2]. The conventional mayonnaise contains 65–80% fat, and its consumption is often linked to health problems [1]. Increased dietary fat, especially saturated fatty acids, is associated with increased risks of high cholesterol, obesity, cardiovascular disease, and hypertension [3]. Obesity is a growing health problem in Romania and in most countries worldwide, and food quality and lifestyle are important factors in maintaining good health and in the prevention of certain ailments [4]. Research shows a concern for the development of new food products such as low-fat mayonnaise like emulsion gel, which is a complex colloidal material stabilized by biopolymers (polysaccharides and proteins) and a low molecular weight emulsifier, in which there are both emulsion droplets and gels [5], and can meet consumer demands, especially for healthy foods. Thus, vegan mayonnaise fortified with carotenoids by the addition of sea buckthorn juice showed improved oxidative stability and did not influence the taste, consistency, or overall acceptability of the mayonnaise [6]. Currently, in food applications, polysaccharides and some proteins, offer advantages in partial replacement of fat droplets to obtain low-fat products, while maintaining textures similar to those of original products. Emulsion gels prepared by simply mixing the alginate solution with egg yolk emulsions, followed by the addition of varying amounts of vinegar to induce gel formation, exhibited significant viscoelasticity and plasticity, comparable to a mayonnaise product containing of 75% fat [7]. Therefore, compared to traditional mayonnaise, emulsion gel has potential applications in fat replacement and as a nutraceutical carrier and has a lower production cost [8].

Oilseed cakes, such as sesame seed cakes and walnut cakes, are by-products of vegetable oil extraction. These by-products, rich in fat, carbohydrates and protein, are suitable after various treatments for incorporation into food products.

Sesame seeds (*Sesamum indicum* L.) occupy an important place in the human diet and can be used as an essential ingredient in various recipes such as salads, pastries, bakery products and in the production of pasta (tahini). Sesame seeds are an important source of oil (44–58%) and show a remarkable stability to oxidation [9]. Industrial processing of sesame seeds generates a large amount of hulls and other waste, which are usually considered a nuisance from a processing point of view. As highlighted in recent work [9,10,11,12,13,14], the potentially beneficial role of the circular economy, an umbrella concept that includes waste minimization and pollution prevention measures, one of the proposed alternative solutions is to recirculate waste in the process, producing value-added products. Defatted sesame flour may contain a protein fraction which is rich in amino acids, and this fraction is considered an attractive protein source from both marketing and formulation points of view, such as addition to food products. In addition, sesame seed cake is rich in lignans, vitamins E, A, the B complex and minerals such as calcium, phosphorus, iron, copper, magnesium, zinc and potassium [14].

Walnuts (*Juglans regia* L.) are a good source of nutrients, especially protein, essential fatty acids, magnesium and manganese, and numerous studies have reported a number of health benefits, such as decreased risk of cardiovascular disease, oxidative stress and cholesterol-lowering effects [15,16]. Partially defatted cake contains polyunsaturated lipids and also a high content of proteins, fiber, minerals, and other bioactive compounds. Residual oil content can vary between 6 and 10%, moisture content can vary between 5.4 and 6.3%, tocopherol content is much lower than in oil, while phenols are more concentrated in cake than in oil [17]. In recent years, there has been a growing demand for natural products in all sectors of life. Thus, walnut seed cake is a renewable and readily available resource, derived mainly from seed waste, and is of great interest as a raw material for the development of food products.

Thus, sesame and walnut cakes are by-products of the oil extraction industry, that still contain many nutritional compounds (polyunsaturated lipids, proteins, fiber, minerals), which deserve to be valued as ingredients for a sustainable diet and food production [18].

Walnut cake, a new product with the potential to be used in the manufacture of biscuits and cakes, was added to macarons by replacing almond flour with 0%, 10%, 25% and 50% powder. The addition of byproduct powder to macarons improved the quality of modified macarons while reducing production costs. [19]. The cakes resulting from the extraction of walnut oil could be used as co-products for high-value-added products, such as topping in bread. To this purpose, Pycia et al. [20] incorporated walnut oil and oil cake (1%, 3%, 5%) as a substitute for wheat flour. The bread obtained was smaller (substitution led to the decrease and weakening of gluten), darker in color, but with a high antioxidant potential compared to the control [20]. The defatted cake obtained after oil extraction was used as a fortification of wheat flour to obtain value-added products such as biscuits [21] or cake [22].

By using new raw materials or unusual flavours in the formulation of food products, which change the complexity of the product, innovative foods can be obtained. Successful innovations can also be the result of a process of searching and combining ingredients from existing recipes [19]. Thus, water-in-oil (W/O) emulsion gels with water, peanut sprout oil, sorbitan monostearate and candelilla wax in different ratios and their potential as substitutes was evaluated of shortening in muffins. In terms of textural properties, it was found that muffins prepared using emulsion gels had a higher hardness than those prepared using fats [23].

The objective of this study was to prepare low-fat mayonnaise-like emulsion gels, with partially defatted sesame and walnuts cakes. Furthermore, the impact of defatted sesame and walnuts cakes supplementation on the physical stability, textural and sensory properties of the end product was also investigated. A commercially available mayonnaise product was used as a reference for comparative purposes.

## 2. Results and Discussion

### 2.1. Emulsion Gels Physicochemical Analysis

Emulsion gels are semi-solid food systems composed of an oil phase dispersed in the gel matrix, macromolecules such as proteins and polysaccharides. By enrichment with different amounts (2 and 4%) of sesame seeds cake (SSC) and walnuts seed cake (WSC) value-added emulsion gels samples were obtained and their physicochemical composition was determined (Table 1).

The characteristics analyzed were compared between the emulsion gel samples obtained and low-fat, egg-free commercial mayonnaise (CM) in an attempt to maintain the characteristics that are most desired by consumers.

The fat content of the samples ranged between 48.86 ± 0.06 and 50.71 ± 0.12, these values were within the range (44.88 ± 1.20 and 60.90 ± 0.57) reported by Amin et al. [24] for sunflower oil mayonnaise samples. Sample M has the lowest fat content of all the samples. It was observed (Table 1) that the fat content of the emulsion gel samples with sesame cake increased to 3.78% for SO2 compared to sample M, while the addition of walnut cake resulted in an increase of 2.08% fat content for WO2, which was lower compared to the fat content determined in the samples with sesame cake.

**Figure 1:**
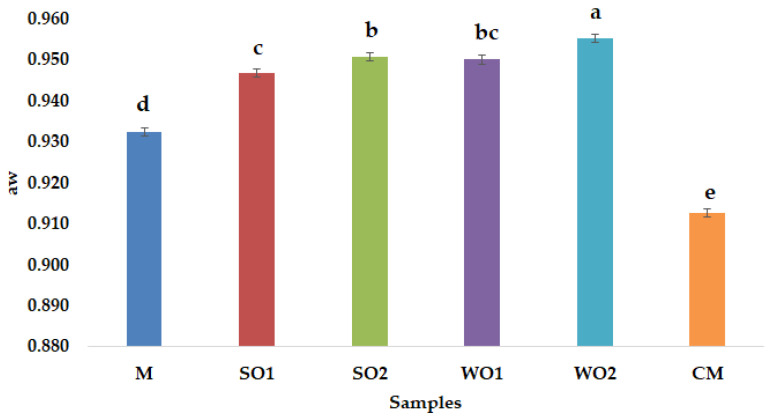
Water activity (aw) of emulsion gel samples: control sample with corn starch (M); samples with 2 and 4% sesame seed cake (SO1 and SO2); samples with 2 and 4% walnut cake (WO1 and WO2); commercial sample (CM). Different lowercase letters indicate the significant differences (*p* < 0.05) among the samples.

**Figure 2:**
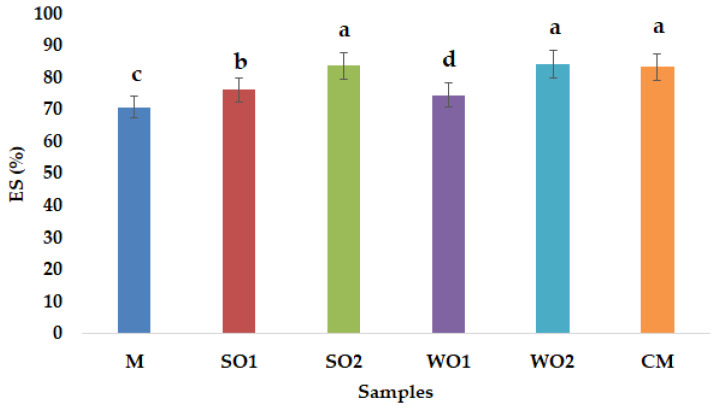
Emulsion stability (ES) index of mayonnaise samples: control sample with corn starch (M); samples with 2 and 4% sesame seed cake (SO1 and SO2); samples with 2 and 4% walnut cake (WO1 and WO2); commercial sample (CM). Different lowercase letters indicate the significant differences (*p* < 0.05) among the samples.

Emulsion gel samples with 4% added sesame seed cake and walnut cake have the highest protein content, 2.86  ±  0.06% and 2.44  ±  0.09% respectively. Proteins play an important role in estimating the nutritional value and food thus in oilseed cakes, the value differs depending on the initial composition of the seeds and the oil yield during pressing [25]. Oilseed cake contains high levels of protein that provides a better balance of amino acids, which are essential for humans, and is attracting more and more attention from food technologists as a functional component [26]. The results showed that fat content increased as SSC and WSC were added, similarly ash content was higher as mayonnaise-like emulsion gels enrichment was achieved. The amount of carbohydrate in the SO1 was found to be higher (19.78 ± 0.49) than that of SO2 (17.50 ± 0.15) on a dry weight basis. The same trend was observed for the walnut cake emulsion gel samples (WO1 and WO2), with the lowest carbohydrate content found for the 4% walnut cake sample (WO2). Furthermore, the caloric value increased by adding SSC and WSC. The caloric values of the emulsion gel samples varied between 523.47 ± 2.27 (WO2) and 546.97 ± 2.92 Kcal/100 g (CM), which were in nearly the same ranges with previous studies [27]. Pintado and Cofrades [28] found that partial replacement of pork fat with oleogel and emulsion gel systems, from chia flour and olive oil, improved the fat lipid profile of four different dry-fermented fuet sausages. Free water in a sample, which influences microbial reproduction, migration and contamination, is indicated by measuring water activity (aw). The quality and safety of mayonnaise-like emulsion gels can be assessed by aw, which values can range between 0.930 (samples containing 77% to 79% oil) and 0.950 (samples containing 37% to 41% oil) according to Ma and Boye [29]. In the present study, all emulsion gel samples that were evaluated had aw values between 0.913 (CM) and 0.955 (WO2) (Figure 1). Only the commercial mayonnaise sample had a lower aw value, the other samples are consistent with the average value which is usually between 0.930 and 0.950 [30]. The aw value of the emulsion gel samples increased (Figure 1), as the amount of added SSC or WSC increased, due to the increased water content of the additives. According to Worrasinchai et al. [31], aw values for mayonnaise samples with partial fat replacement by β-glucan ranged from 0.989 to 0.998, while Khushbu and Sunil [32] reported that mayonnaise samples with refined sunflower oil, full fat (80.5%) and low fat (60.0%) had the following aw values: 0.880 and 0.940, respectively. Figure 2 shows the emulsion stability index values obtained for the emulsion gel samples, which are close to those reported by Park et al. [33]. The emulsion stability (ES) of emulsion gel samples is influenced by the particle size of the dispersed phase, the difference in density between the oil and water phases, the viscosity of the emulsion and it is well known that mayonnaise with a higher amount of oil is more stable [34]. Emulsifying agents that can replace egg yolk in oil/water emulsions are whey protein, wheat gluten, soy protein and corn starch [35].

SSC and WSC were introduced as a replacement of egg yolk, by gelling with gelatin to obtain emulsion gels like low-cholesterol mayonnaise with the same textural properties as the conventional ones. Vegetable protein is considered a sustainable alternative for animal protein, but there are differences in emulsification and gelation properties on the structure and performance of emulsion gels [36]. Moreover, when the added ratio oil seeds cake was 4%, the emulsion stability index of emulsion gel increased from 70.73% to 83.64%, which may be explained by the coagulation capacity of an SSC paste added. Sample with 4% walnut cake (WO2) has also a high emulsion stability index (84.09%), not statistically different that of CM sample. Park et al. [33] reported emulsion stability of 86.78 and 85.14% for low-fat mayonnaise (fat replacement ratio of 30% and 50%, respectively). Drozłowska et al. [37] reported that in the case of emulsion stabilization, flaxseed oil cake extract (FOCE) were capable to stabilize O/W emulsions. In similar work with a camelina press-cake, Burgos-Díaz et al. [38] indicated a remarkable emulsifying capacity at a concentration of ≥2.5%, *w*/*w* and that potentially coarse protein mixtures have good emulsifying properties.

**Figure 3:**
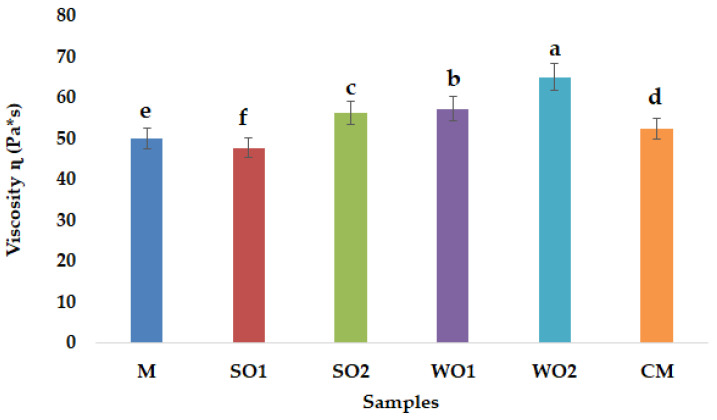
Viscosity ɳ (Pa*s) of mayonnaise-like emulsion gel samples: control sample with corn starch (M); samples with 2 and 4% sesame seed cake (SO1 and SO2); samples with 2 and 4% walnut cake (WO1 and WO2); commercial sample (CM). Different lowercase letters indicate the significant differences (*p* < 0.05) among the samples.

The viscosity is the tendency of a liquid to resist flow under stress [39]. Results presented in Figure 3 reveal that the concentration affected the apparent viscosity for all emulsion gels samples compared to the commercial sample. All samples show a significant difference from the control sample (M) (*p* < 0.05). The apparent viscosity values at 20 s^−1^ and 20 °C for different emulsion gels samples ranged from 65.0 to 47.73 (Pa*s) with the highest value for formula WO2 (emulsion gel with 4% WSC) and the lowest value for formula SO1 (emulsion gel with 2% SSC). The type and concentration of oil seeds cake affected the apparent viscosity of the formulation. According to Hosseini et al. [40], gum arabic concentration and storage temperature had a significant effect on emulsion viscosity, by increasing the gum concentration from 2% to 8%, the emulsion viscosity was drastically increased. Viscosity changes could be related to the balance of chemical forces of interparticle colloidal attraction and repulsion [41].

**Figure 4:**
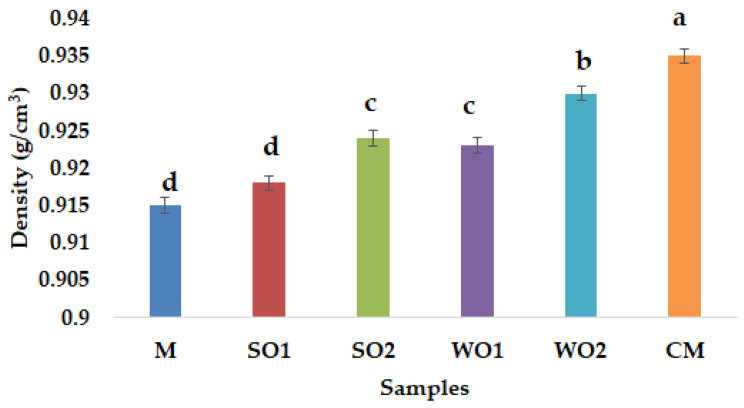
Density (g/cm^3^) of emulsion gel samples: control sample with corn starch (M); samples with 2 and 4% sesame seed cake (SO1 and SO2); samples with 2 and 4% walnut cake (WO1 and WO2); commercial sample (CM). Different lowercase letters indicate the significant differences (*p* < 0.05) among the samples.

According to Figure 4, the amount of sesame cake and walnut cake added has an increasing effect on the density of the mayonnaise-like emulsion gel samples, thus, the highest measured value was for the WO2 formula (gel emulsion with 4% WSC). In the case of emulsions, a destabilization phenomenon, known as creaming, can be observed, resulting from the migration of oil droplets in the upper part of the sample due to density differences with the continuous phase. In general, the results obtained revealed the macroscopic stability of the emulsions gels samples over the tested period of 5 days. Lastra-Ripoll et al. [42] demonstrated that sesame seed cake contains hydrocolloids that can be used as emulsifying agents, while Shi et al. [43] investigated the emulsion capacity of walnut protein.

Consumer acceptance of food is largely influenced by color [44]. Using unconventional ingredients to enhance the final product, different from those used in a standard recipe, changes the color [44]. Table 2 shows the results of the color measurements obtained for the low-fat mayonnaise-like emulsion gel samples. Lightness index (L*) values of emulsion gel samples decreased when corn starch was substituted with oil seed cakes, the color of these samples being darker than CM and M samples. The low-fat mayonnaise-like emulsion gel samples had a yellow–green color (a* values situated in the negative region more toward green and b* values situated in the positive region more toward yellow). Color parameters are highly influenced by the ingredients. Malaviya and Yadav [45], investigated the color of cake sesame seeds and determined that L* had values ranging from 33.39 to 65.49, while a* values ranged from 0.20 to 3.33, and the b* parameter had values ranging from 2.32 to 12.41. Due to the loss of colored oil during extraction, sesame seed cake has higher color parameter values than sesame seeds, which can have color from black to white due to the presence or absence of certain genes [45]. Walnut kernel color can be golden, dark gold and brown [46], while walnut oilcake color varies from yellow to light brown. Pigments like chlorophylls and carotenoids were found in walnut virgin oil, and they mainly contribute to the color of the oil (L*: 42.8–47.0; a*: −0.5 to −2.1; b*: 10.5–13.9) [15]. According to research by Hadnađev et al. [47] on the results of color measurement of confectionery fillings containing maltodextrin gel portion, formulations including fat replacers were darker than the control sample. Successive increase in amount of both maltodextrin types resulted in gradual decrease in L* values [47].

### 2.2. Low-Fat Mayonnaise-like Emulsion GelsTexture Profile Analysis

The average values of hardness, adhesiveness, springiness, cohesiveness, gumminess and chewiness for each sample was presented in Table 3. It was observed that hardness, adhesiveness, gumminess, chewiness decreased with the increase of oil cake content added, while springiness and cohesiveness increased. This trend was observed by Wang et al. [48] which substituted the egg yolk with soybean oil body in mayonnaise and concluded that the hardness and adhesiveness of the mayonnaise decreased with the increase of soybean oil body substitution ratio.

The hardness of commercial mayonnaise (CM) was 6.12 ± 0.03 N, while the control lab sample (M) recorded a hardness value of 4.8 ± 0.03 N (Table 3). When the substitution of corn starch with sesame seed cake increased, the hardness of substituted mayonnaise decreased with 79.16%, while springiness increased with 49.25%. Also, adhesiveness decreased with 79.51%, and cohesiveness increased with 23.33%.

In the case of corn starch substitution with walnut seed cake (4%), it was observed that the hardness of substituted mayonnaise decreased with 76.66%, springiness increased with 65.67%, while adhesiveness decreased with 77.65% and cohesiveness increased with 20.00%.

### 2.3. Sensory Quality Attributes of Low-Fat Mayonnaise-like Emulsion Gel Samples

**Figure 5:**
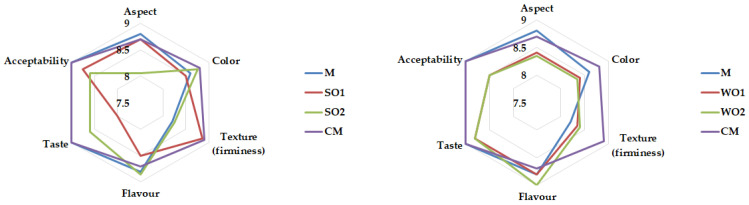
Sensory evaluation of emulsion gel samples: control sample with corn starch (M); samples with 2 and 4% sesame seed cake (SO1 and SO2); samples with 2 and 4% walnut cake (WO1 and WO2); commercial sample (CM).

Emulsion gel samples were assessed by a group of twenty panelists. The average scores of selected attributes (aspect, color, texture/firmness, flavor, taste and acceptability) are illustrated in Figure 5. All panelists highly appreciated (‘like very much’ to ‘like extremely’) aspect and texture of SO1, and color, flavor and taste of SO2. The highest scores were obtained for the following sensory attributes: texture (8.85 ± 0.02, for SO1) and flavor (8.85 ± 0.10 for SO2), while the lowest average was determined for taste (8.00 ± 0.01 for SO2) and aspect (8.05 ± 0.03 for SO2). Samples WO1 and WO2 being liked very much to liked extremely especially for taste (8.80 ± 0.01), flavor (8.80 ± 0.04), aspect (8.40 ± 0.01), and color (8.40 ± 0.02) (WO1), and for flavor (8.89 ± 0.01), taste (8.80 ± 0.20) and texture (8.40 ± 0.30) (WO2). The panelists highly appreciated the flavor and taste of emulsion gels with walnut seed cake, and color of emulsion gels with sesame seeds cake. Some carbohydrate-based fat replacers (potato maltodextrin and specially derived waxy corn maltodextrin) that form water-in-oil (W/O) emulsion gels have been evaluated in confectionery fillings. The results according to the product acceptance-preference test show that the confectionery filling with 5 g/100 g of fat had the highest scores, but the product with 15 g/100 g of fat was also sensory acceptable [47].

### 2.4. Principal Component Analysis

**Figure 6:**
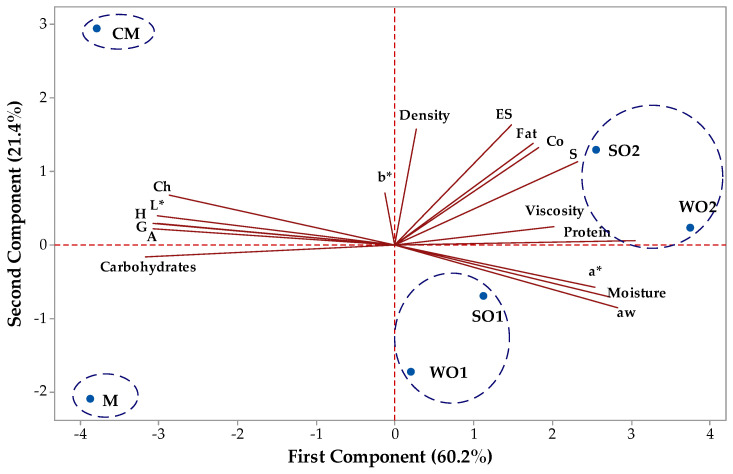
Biplot of scores and loadings of data obtained from physico-chemical parameters (fat, protein, moisture, carbohydrate, aw, emulsion stability (ES), viscosity, density), color parameters (L*—lightness; a*—greenness; b*—yellowness) and texture attributes (hardness (H), adhesiveness (A), springiness (S), cohesiveness (Co), gumminess (G), chewiness (Ch) of emulsion gel samples (control sample with corn starch (M); samples with 2 and 4% sesame seed cake (SO1 and SO2); samples with 2 and 4% walnut cake (WO1 and WO2); commercial sample (CM).

Principal component analysis (PCA) was used to observe if commercial and prepared emulsion gels can be grouped and classified based on their similarities and differences of their characteristics. Also, the interrelationships among variables/characteristics are revealed with PCA. Principal component (PC1) had an eigenvalue of 10.23 and explained 60.2% of the total variation, while the second component (PC2) had an eigenvalue of 3.64 and accounted for 21.4% of the total variation. The first two PC took into account 81.6% of the total variation. A biplot, illustrated in Figure 6, which combined scores and loadings plot for two components was considered for the entire data set visualization. From Figure 6 it can be observed that parameters like L* (−0.300), gumminess (−0.300), hardness (−0.295), adhesiveness (−0.295), chewiness (−0.280), carbohydrate (−0.309) of low-fat mayonnaise-like emulsion gel samples had negative loadings on the PC1, while the other parameters like protein (0.299), aw (0.277), moisture (0.267), a* (0.248) and springiness (0.227) of mayonnaise samples had positive loadings. The factor loadings represent the correlation between the physico-chemical, color parameters and texture attributes. PC2 show a negative correlation with aw (−0.235), a* (−0.156), moisture (−0.194) and carbohydrates (−0.042) and positive correlation with the other parameters (higher correlations with ES (0.452), density (0.435) and fat (0.382)). Figure 6 shows that two mayonnaises (control sample with corn starch (M) and commercial sample (CM) were situated to the left in the score biplot and had negative values for PC1, while low-fat mayonnaise-like emulsion gel samples with sesame seed cake (SO1 and SO2) and walnut cake (WO1 and WO2) were located at the right in the score biplot and had positive values for PC1. SO2 and WO2 low-fat mayonnaise-like emulsion gel samples had higher positive values for PC1 than SO1 and WO1.

## 3. Conclusions

The main goal of the paper was to prepare low-fat vegan mayonnaise-like emulsion gels (W/O) with different concentrations of by-products from oil extraction, sesame seed cakes and walnut cakes. The fat content of the emulsion gel samples was lower than that of commercial mayonnaise (CM), with one exception (sample with 2% SSC added). All samples had protein content compared to commercial mayonnaise and lower calorie content. The replacement of corn starch with oil cake showed an increase in emulsion stability, while the samples with the addition of 2% SSC and WSC show an emulsion stability index that is not statistically different from that of commercial mayonnaise. Increasing the amount of oil cake from 2 to 4 g/100 g resulted in a decrease in L* parameter values, and it was observed that low fat systems were darker. With increasing SSC addition, the hardness of low-fat mayonnaise-like emulsion gels decreased with 79.16%, while springiness increased with 49.25%. In emulsion preparation, with WSC, it was observed that the springiness and cohesiveness of emulsion gels increased with the increase of WSC concentration, but the hardness presented the opposite trend. Walnut cake emulsion gels were highly appreciated for their flavor and taste, while sesame seed cake emulsion gels were more appreciated for the color. Technologically, this recipe change can be allowed without significantly changing the recipe, but market testing will be required. Consequently, further extended studies should be carried out, for example, on the microbial properties during storage of emulsion gels or the sensory perception of new formulations with the addition of natural dyes, herb and spice extracts and antioxidants in emulsion gels, to promote their use in food.

## 4. Materials and Methods

### 4.1. Materials

Emulsion gel samples were produced in the faculty laboratory from sesame and walnut cakes obtained after cold-pressing the seeds (oil extraction) and grinding them with a blender (to obtain homogeneous flour). Sesame seeds and walnuts were purchased online from organic stores. Sunflower oil, corn starch, mustard (used as an emulsifier to stabilize the emulsion), lemon, sugar, gelatin and salt were purchased from local markets.

### 4.2. Preparation of Mayonnaise-like Emulsion Gels

**Figure 7:**
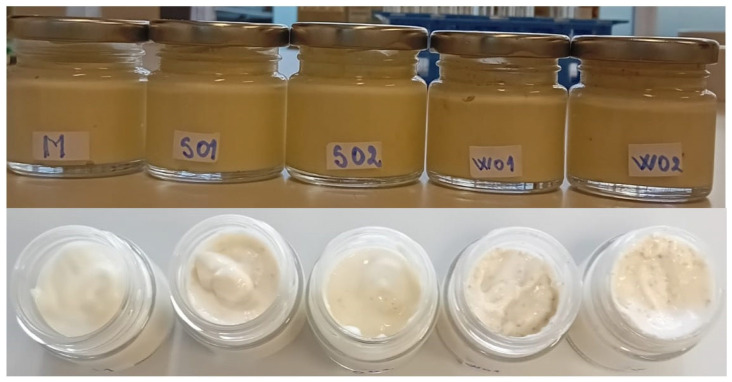
Mayonnaise-like emulsion gel samples: control sample with corn starch (M); samples with 2 and 4% sesame seed cake (SO1 and SO2); samples with 2 and 4% walnut cake (WO1 and WO2).

The emulsions were prepared according to Burgos-Díaz et al. [38] with some modifications. The low-fat mayonnaise-like emulsion gels were prepared in three steps: (i) Gelatin granules (4.0%, *m*/*v*) were dispersed in ultrapure water at room temperature. After 30 min, they were treated at 45 °C for 30 min in an, constant-temperature water bath (Model Precisterm, JP, Selecta, 5 l, Barcelona, Spain). The diluted gelatin solution 1.0% was obtained by diluting 4.0% gelatin solution with ultrapure water [49] the aqueous phase and water-soluble ingredients (press cake flour, sugar, salt and mustard) were mixed together with a Homogenize OV5, VELP, (Scientifica, Usmate, Italy); (ii) the small fraction of the total sunflower oil was added slowly for 1 min during mixing, and the acid phase (lemon juice) was added after mixing for another 60 s; (iii) the remaining major amount of sunflower oil was added slowly and mixed [50]. The obtained emulsion gels samples were stored at 4 °C (Figure 7).

The formulations of the emulsion gels samples prepared for this study are presented in Table 4. To investigate the effect of press cake types on emulsion gel development, emulsion gel products containing 2 and 4%, respectively, were prepared by a similar procedure. The percentages of 2 and 4% added press cake flour, were chosen after a pre-experiment conducted to form emulsion gels samples with 2–6% added cake.

### 4.3. Physicochemical Analysis

The chemical properties (moisture, fat, ash) of samples were analyzed according to standard methods [51]. Proteins were calculated from the nitrogen content determined through the Kjeldahl method using the conversion factor 6.38.

The total carbohydrate content was calculated by the difference:

[100 − (protein + lipids + ash + moisture)],(1)

Caloric value of the mayonnaise-like emulsion gel sample was calculated as per [48]:

Caloric values (Kcal/100 g) = (4 × protein) + (9 × fat) + (4 × carbohydrate),(2)

The proximate composition of the sesame cake fractions was determined and reported in previous work [52].

Samples of emulsion gels were evaluated for water activity (aw) through the water activity meter AquaLab Lite (Decagon Devices, Inc., Washington, USA), at room temperature.

The density of the emulsion gels samples was measured using the pycnometer according to the method described by [53].

Centrifugal stability of emulsion gels samples was determined as follows: 8 g (F0) of the emulsion gel sample were transferred to the centrifuge tube and centrifuged for 15 min at 3000 rpm [27]. The remaining emulsion layer (F1) was measured, and the emulsion stability was calculated according to Equation (3) [37]:

Emulsion stability (%) = (F1/F0) × 100,(3)

Viscosity measurements of the emulsion gels were performed using a Brookfield viscometer (Brookfield Engineering Inc., Model RV-DV II Pro+, Middleborough, MA, USA). The results were expressed in Pa·s.

Color was measured in terms of L* (lightness/darkness), a* (redness/greenness), and b* (yellowness/blueness) values, using a Minolta Chroma Meter (Model CR 310, Minolta Camera Co. Ltd., Tokyo, Japan), [52].

### 4.4. Textural Analysis

To evaluate hardness (H), adhesion (A), springiness (S), cohesiveness (Co), chewiness (Ch) and gumminess (G) index of the emulsion gels samples, a laboratory texturometer MARK–10 Force Gauge Model M 5–20 (Mark-10 Corporation, Copiague, NY, USA) was used.

### 4.5. Sensory Evaluation

The sensory analysis was conducted with twenty semi-trained panelists of both sexes, randomly selected among students and professors of the Faculty of Food Engineering, Stefan cel Mare University of Suceava, Romania. Sensory attributes of coded low-fat mayonnaise-like emulsion gel samples were conducted at room temperature (approximately 20 °C), placed on white china plates. To neutralize the taste, the commission used the middle part of salt cookie and tepid lemon-flavored water (concentration 1%) [54]. The panelists evaluated the sensory characteristics (aspect, color, texture, flavor, taste, and overall acceptability) of emulsion gels [24]. The analysis was performed by scoring sensory attributes by assigning a scale from 1 to 9 points (1 = lowest score and 9 = highest score). The data were adjusted to the average score assigned to each attribute by each panelist.

### 4.6. Statistical Analysis

The experiments were performed in triplicate and the obtained results were expressed as mean values ± standard mean error. Minitab version 17 (Minitab, Inc., State College, PA, USA) was used for statistical evaluation. An analysis of variance (ANOVA) with a 95% confidence interval (*p* < 0.05) and Tukey’s test were considered to compare the results obtained. In addition, a principal components analysis was carried out.

## Figures and Tables

**Table 1:** Proximate composition (g/100 g) of emulsion gel samples: control sample with corn starch (M); samples with 2 and 4% sesame seed cake (SO1 and SO2); samples with 2 and 4% walnut cake (WO1 and WO2); commercial sample (CM).

Samples	Fat (%)	Protein (%)	Ash (%)	Moisture (%)	Carbohydrate (%)	Caloric Value (Kcal/100 g)
M	48.86 ± 0.06 ^c^	0.58 ± 0.15 ^e^	0.36 ± 0.02 ^e^	26.24 ± 0.09 ^de^	23.96 ± 0.12 ^a^	537.90 ± 0.53 ^b^
SO1	49.79 ± 0.49 ^abc^	1.75 ± 0.04 ^c^	0.94 ± 0.02 ^c^	27.74 ± 0.02 ^b^	19.78 ± 0.49 ^b^	534.21 ± 2.60 ^b^
SO2	50.71 ± 0.12 ^a^	2.86 ± 0.06 ^a^	2.06 ± 0.06 ^b^	26.86 ± 0.06 ^cd^	17.50 ± 0.15 ^c^	537.83 ± 0.93 ^b^
WO1	49.64 ± 0.64 ^bc^	1.46 ± 0.06 ^d^	0.54 ± 0.03 ^d^	27.54 ± 0.03 ^bc^	20.82 ± 0.61 ^b^	535.86 ± 3.09 ^b^
WO2	49.88 ± 0.14 ^ab^	2.44 ± 0.09 ^b^	2.91 ± 0.12 ^a^	28.58 ± 0.49 ^a^	16.19 ± 0.24 ^d^	523.47 ± 2.27 ^c^
CM	50.05 ± 0.23 ^ab^	0.32 ± 0.03 ^f^	0.24 ± 0.04 ^e^	25.58 ± 0.49 ^e^	23.81 ± 0.42 ^a^	546.97 ± 2.92 ^a^

Values are mean ± standard deviation (n = 3). Means that do not share a letter within a column are significantly different (*p* ≤ 0.05).

**Table 2:** Color parameters of low-fat mayonnaise-like emulsion gel samples: control sample with corn starch (M); samples with 2 and 4% sesame seed cake (SO1 and SO2); samples with 2 and 4% walnut cake (WO1 and WO2); commercial sample (CM).

Samples	L*	a*	b*
M	65.81 ± 0.10 ^b^	−6.75 ± 0.04 ^c^	15.89 ± 0.06 ^d^
SO1	59.94 ± 0.24 ^c^	−4.32 ± 0.04 ^b^	18.19 ± 0.03 ^b^
SO2	56.60 ± 0.06 ^e^	−4.30 ± 0.03 ^b^	19.83 ± 0.03 ^a^
WO1	57.30 ± 0.13 ^d^	−1.37 ± 0.02 ^a^	14.18 ± 0.15 ^e^
WO2	52.31 ± 0.42 ^f^	−1.30 ± 0.02 ^a^	13.18 ± 0.11 ^f^
CM	66.85 ± 0.16 ^a^	−6.95 ± 0.04 ^d^	16.89 ± 0.06 ^c^

Values are mean ± standard deviation (n = 3). Means that do not share a letter within a column are significantly different (*p* ≤ 0.05).

**Table 3:** Texture attributes of low-fat mayonnaise-like emulsion gel samples: control sample with corn starch (M); samples with 2 and 4% sesame seed cake (SO1 and SO2); samples with 2 and 4% walnut cake (WO1 and WO2); commercial sample (CM).

Samples	Texture Attributes					
	Hardness (N)	Adhesiveness (Joule)	Springiness (mm)	Cohesiveness	Gumminess (N)	Chewiness (N*mm)
M	4.8 ± 0.03 ^b^	16.11 ± 0.04 ^b^	0.67 ± 0.01 ^d^	0.6 ± 0.03 ^b^	3.00 ± 0.04 ^b^	2.00 ± 0.03 ^b^
SO1	1.02 ± 0.04 ^e^	4.00 ± 0.03 ^d^	0.94 ± 0.02 ^bc^	0.74 ± 0.02 ^a^	0.76 ± 0.03 ^d^	0.71 ± 0.04 ^e^
SO2	1.00 ± 0.03 ^e^	3.3 ± 0.04 ^f^	1.00 ± 0.01 ^b^	0.74 ± 0.02 ^a^	0.6 ± 0.02 ^e^	0.84 ± 0.03 ^d^
WO1	2.6 ± 0.01 ^c^	10.88 ± 0.02 ^c^	0.74 ± 0.03 ^d^	0.65 ± 0.03 ^b^	1.69 ± 0.02 ^c^	1.25 ± 0.02 ^c^
WO2	1.12 ± 0.03 ^d^	3.6 ± 0.03 ^e^	1.11 ± 0.03 ^a^	0.72 ± 0.01 ^a^	0.65 ± 0.01 ^e^	0.85 ± 0.02 ^d^
CM	6.12 ± 0.03 ^a^	20.6 ± 0.04 ^a^	0.91 ± 0.04 ^c^	0.72 ± 0.02 ^a^	3.65 ± 0.02 ^a^	2.85 ± 0.03 ^a^

Values are mean ± standard deviation (n = 3). Means that do not share a letter within a column are significantly different (*p* ≤ 0.05).

**Table 4:** Formulation of analyzed mayonnaise-like emulsion gel samples (wt%).

Ingredients	Samples				
	M	SO1	SO2	WO1	WO2
Corn starch	4	-	-	-	-
Sesame seeds cake (SSC)	-	2	4	-	-
Walnuts cake (WSC)	-	-	-	2	4
Lemon juice	7	7	7	7	7
Sunfloweroil	37	39	37	39	37
Mustard	1.5	1.5	1.5	1.5	1.5
Sugar	1	1	1	1	1
Salt	1.5	1.5	1.5	1.5	1.5
Gelatin	2	2	2	2	2
Water	46	46	46	46	46

## Data Availability

All data and materials are available on request from the corresponding author. The data are not publicly available due to ongoing researches using a part of the data.

## References

[1] Mirzanajafi-Zanjani M., Yousefi M., Ehsani A. (2019). Challenges and approaches for production of a healthy and functional mayonnaise sauce. Food Sci. Nutr..

[2] Di Mattia C., Balestra F., Sacchetti G., Neri L., Mastrocola D., Pittia P. (2015). Physical and structural properties of extra-virgin olive oil based mayonnaise. LWT-Food Sci. Technol..

[3] Senila L., Neag E., Cadar O., Kovacs M.H., Becze A., Senila M. (2020). Chemical, nutritional and antioxidant characteristics of different food seeds. Appl. Sci..

[4] Mititelu M., Oancea C.-N., Neacșu S.M., Musuc A.M., Gheonea T.C., Stanciu T.I., Rogoveanu I., Hashemi F., Stanciu G., Ioniță-Mîndrican C.-B. (2023). Evaluation of Junk Food Consumption and the Risk Related to Consumer Health among the Romanian Population. Nutrients.

[5] Lin D., Kelly A.L., Miao S. (2020). Preparation, structure-property relationships and applications of different emulsion gels: Bulk emulsion gels, emulsion gel particles, and fluid emulsion gels. Trends Food Sci. Technol..

[6] Nour V. (2021). Effect of sea buckthorn juice addition on the oxidative stability, physicochemical and sensory properties of soy milk mayonnaise during refrigerated storage. Ukr. J. Food Sci..

[7] Yang X., Li A., Yu W., Li X., Sun L., Xue J., Guo Y. (2020). Structuring oil-in-water emulsion by forming egg yolk/alginate complexes: Their potential application in fabricating low-fat mayonnaise-like emulsion gels and redispersible solid emulsions. Int. J. Biol. Macromol..

[8] Song L., Zhang S., Liu B. (2022). The fabrication and characterization of Pickering emulsion gels stabilized by sorghum flour. Foods.

[9] Elleuch M., Besbes S., Roiseux O., Blecker C., Attia H. (2007). Quality characteristics of sesame seeds and by-products. Food Chem..

[10] Fidelis M., de Moura C., Kabbas Junior T., Pap N., Mattila P., Mäkinen S., Putnik P., Bursać Kovačević D., Tian Y., Yang B. (2019). Fruit Seeds as Sources of Bioactive Compounds: Sustainable Production of High Value-Added Ingredients from By-Products within Circular Economy. Molecules.

[11] Cozma P., Smaranda C., Comăniță E.D., Roşca M., Ghinea C., Campean T., Gavrilescu M. (2020). Knowledge transfer in university-industry research collaboration for extending life cycle of materials in the context of circular economy. Environ. Eng. Manag. J..

[12] Teo S.H., Ching Y.C., Fahmi M.Z., Lee H.V. (2023). Surface Functionalization of Sugarcane-Bagasse-Derived Cellulose Nanocrystal for Pickering Emulsion Gel: Microstructural Properties and Stability Efficiency. Gels.

[13] Petraru A., Ursachi F., Amariei S. (2021). Nutritional characteristics assessment of sunflower seeds, oil and cake. Perspective of using sunflower oilcakes as a functional ingredient. Plants.

[14] Wei P., Zhao F., Wang Z., Wang Q., Chai X., Hou G., Meng Q. (2022). Sesame (*Sesamum Indicum* L.): A comprehensive review of nutritional value, phytochemical composition, health benefits, development of food, and industrial applications. Nutrients.

[15] Leahu A., Oroian M., Ropciuc S. (2016). The quality and stability of walnut oil under the influence of storage conditions. Sci. Pap. Ser. Anim. Sci..

[16] Petrović-Oggiano G., Debeljak-Martačić J., Ranković S., Pokimica B., Mirić A., Glibetić M., Popović T. (2020). The Effect of Walnut Consumption on *n*-3 Fatty Acid Profile of Healthy People Living in a Non-Mediterranean West Balkan Country, a Small Scale Randomized Study. Nutrients.

[17] Ojeda-Amador R.M., Salvador M.D., Gómez-Alonso S., Fregapane G. (2018). Characterization of virgin walnut oils and their residual cakes produced from different varieties. Food Res. Int..

[18] Smeu I., Dobre A.A., Cucu E.M., Mustățea G., Belc N., Ungureanu E.L. (2022). Byproducts from the vegetable oil industry: The challenges of safety and sustainability. Sustainability.

[19] Pop A., Păucean A., Socaci S.A., Alexa E., Man S.M., Mureșan V., Chiş M.S., Salanță L., Popescu I., Berbecea A. (2020). Quality characteristics and volatile profile of macarons modified with walnut oilcake by-product. Molecules.

[20] Pycia K., Kapusta I., Jaworska G. (2020). Walnut oil and oilcake affect selected the physicochemical and antioxidant properties of wheat bread enriched with them. J. Food Process. Preserv..

[21] Prakash K., Naik S.N., Vadivel D., Hariprasad P., Gandhi D., Saravanadevi S. (2018). Utilization of defatted sesame cake in enhancing the nutritional and functional characteristics of biscuits. J. Food Process. Preserv..

[22] Melo D., Álvarez-Ortí M., Nunes M.A., Costa A.S., Machado S., Alves R.C., Pardo J.E., Oliveira M.B.P. (2021). Whole or defatted sesame seeds (*Sesamum indicum* L.)? The effect of cold pressing on oil and cake quality. Foods.

[23] Jeong H., Huh C.K., Ha H.K., Kim J., Oh I. (2023). Development of an Emulsion Gel Containing Peanut Sprout Oil as a Fat Replacer in Muffins: Physicochemical, Tomographic, and Texture Properties. Gels.

[24] Amin M.H.H., Elbeltagy A.E., Mustafa M., Khalil A.H. (2014). Development of low-fat mayonnaise containing different types and levels of hydrocolloid gum. J. Agroaliment. Process. Technol..

[25] Rani R., Badwaik L.S. (2021). Functional properties of oilseed cakes and defatted meals of mustard, soybean and flaxseed. Waste Biomass Valorization.

[26] Kotecka-Majchrzak K., Sumara A., Fornal E., Montowska M. (2020). Oilseed proteins–Properties and application as a food ingredient. Trends Food Sci. Technol..

[27] Pintado T., Herrero A.M., Jiménez-Colmenero F., Ruiz-Capillas C. (2016). Emulsion gels as potential fat replacers delivering β-glucan and healthy lipid content for food applications. J. Food Sci. Technol..

[28] Pintado T., Cofrades S. (2020). Quality characteristics of healthy dry fermented sausages formulated with a mixture of olive and chia oil structured in oleogel or emulsion gel as animal fat replacer. Foods.

[29] Ma Z., Boye J.I. (2013). Advances in the Design and Production of Reduced-Fat and Reduced-Cholesterol Salad Dressing and Mayonnaise: A Review. Food Bioprocess Technol..

[30] Carcelli A., Crisafulli G., Carini E., Vittadini E. (2020). Can a physically modified corn flour be used as fat replacer in a mayonnaise?. Eur. Food Res. Technol..

[31] Worrasinchai S., Suphantharika M., Pinjai S., Jamnong P. (2006). β-Glucan prepared from spent brewer’s yeast as a fat replacer in mayonnaise. Food Hydrocoll..

[32] Khushbu S., Sunil C.K. (2018). Comparative study on effect of shallot flour as a thickener, with commercially available thickeners on properties of low fat mayonnaise. Trends Biosci..

[33] Park J.J., Olawuyi I.F., Lee W.Y. (2020). Characteristics of low-fat mayonnaise using different modified arrowroot starches as fat replacer. Int. J. Biol. Macromol..

[34] Kumar Y., Roy S., Devra A., Dhiman A., Prabhakar P.K. (2021). Ultrasonication of mayonnaise formulated with xanthan and guar gums: Rheological modeling, effects on optical properties and emulsion stability. LWT.

[35] Herald T.J., Abugoush M., Aramouni F. (2009). Physical and sensory properties of egg yolk and egg yolk substitutes in a model mayonnaise system. J. Texture Stud..

[36] Sagis L.M., Yang J. (2022). Protein-stabilized interfaces in multiphase food: Comparing structure-function relations of plant-based and animal-based proteins. Curr. Opin. Food Sci..

[37] Drozłowska E., Bartkowiak A., Łopusiewicz Ł. (2020). Characterization of flaxseed oil bimodal emulsions prepared with flaxseed oil cake extract applied as a natural emulsifying agent. Polymers.

[38] Burgos-Díaz C., Mosi-Roa Y., Opazo-Navarrete M., Bustamante M., Garrido-Miranda K. (2022). Comparative Study of Food-Grade Pickering Stabilizers Obtained from Agri-Food Byproducts: Chemical Characterization and Emulsifying Capacity. Foods.

[39] Pourramezan H., Labbafi M., Khodaiyan F., Mousavi M., Gharaghani M., Saadatvand M., Mahmoudi A. (2022). Preparation of octenyl succinylated kappa-carrageenan; reaction optimization, characterization, and application in low-fat vegan mayonnaise. Int. J. Biol. Macromol..

[40] Hosseini A., Jafari S.M., Mirzaei H., Asghari A., Akhavan S. (2015). Application of image processing to assess emulsion stability and emulsification properties of Arabic gum. Carbohydr. Polym..

[41] Golchoobi L., Alimi M., Shokoohi S., Yousefi H. (2016). Interaction between nanofibrillated cellulose with guar gum and carboxy methyl cellulose in low-fat mayonnaise. J. Texture Stud..

[42] Lastra-Ripoll S.E., Quintana S.E., Garcia-Zapateiro L.A. (2022). Chemical, technological, and rheological properties of hydrocolloids from sesame (*Sesamum indicum*) with potential food applications. Arab. J. Chem..

[43] Shi L.S., Yang X.Y., Gong T., Hu C.Y., Shen Y.H., Meng Y.H. (2023). Ultrasonic treatment improves physical and oxidative stabilities of walnut protein isolate-based emulsion by changing protein structure. LWT.

[44] Flamminii F., Di Mattia C.D., Sacchetti G., Neri L., Mastrocola D., Pittia P. (2020). Physical and sensory properties of mayonnaise enriched with encapsulated olive leaf phenolic extracts. Foods.

[45] Malaviya R., Yadav N. (2022). Exploring Nutritional and Functional Properties of Different Varieties of Sesame Seed Cakes: An Industrial By-Product. Plant Arch..

[46] Iordănescu O.A., Radulov I., Buhan I.P., Cocan I., Berbecea A.A., Popescu I., Poșta D.S., Camen D., Lalescu D. (2021). Physical, Nutritional and Functional Properties of Walnuts Genotypes (*Juglans regia* L.) from Romania. Agronomy.

[47] Hadnađev M., Hadnađev T.D., Dokić L., Pajin B., Torbica A., Šarić L., Ikonić P. (2014). Physical and sensory aspects of maltodextrin gel addition used as fat replacers in confectionery filling systems. LWT-Food Sci. Technol..

[48] Wang W., Hu C., Sun H., Zhao J., Xu C., Ma Y., Ma J., Jiang L., Hou J. (2022). Physicochemical Properties, Stability and Texture of Soybean-Oil-Body-Substituted Low-Fat Mayonnaise: Effects of Thickeners and Storage Temperatures. Foods.

[49] Ding M., Zhang T., Zhang H., Tao N., Wang X., Zhong J. (2020). Gelatin-stabilized traditional emulsions: Emulsion forms, droplets, and storage stability. Food Sci. Hum. Wellness.

[50] Yang X., Gong T., Li D., Li A., Sun L., Guo Y. (2019). Preparation of high viscoelastic emulsion gels based on the synergistic gelation mechanism of xanthan and konjac glucomannan. Carbohydr. Polym..

[51] FAO Chapter 4: Summary—Integration of Analytical Methods and Food Energy Conversion Factors. https://www.fao.org/3/y5022e/y5022e05.htm#bm5.

[52] Leahu A., Ghinea C., Petraru A., Ropciuc S. Defatted Sesame Seed Cake: Influence on the Physicochemical and Textural Characteristics of Mayonnaise. Proceedings of the 2022 E-Health and Bioengineering Conference (EHB).

[53] Ozcan I., Ozyigit E., Erkoc S., Tavman S., Kumcuoglu S. (2023). Investigating the physical and quality characteristics and rheology of mayonnaise containing aquafaba as an egg substitute. J. Food Eng..

[54] Karas R., Skvarča M., Žlender B. (2002). Sensory quality of standard and light mayonnaise during storage. Food Technol. Biotechnol..

